# 9-Chloro-1-methyl-7-phenyl-5,6-dihydro-13*H*-indolo[3,2-*c*]acridine

**DOI:** 10.1107/S1600536809013737

**Published:** 2009-04-18

**Authors:** Makuteswaran Sridharan, Karnam J. Rajendra Prasad, Matthias Zeller

**Affiliations:** a Department of Chemistry, Bharathiar University, Coimbatore 641 046, Tamil Nadu, India; b Department of Chemistry, Youngstown State University, One University Plaza, Youngstown, OH 44555, USA

## Abstract

The title compound, C_26_H_19_ClN_2_, is a 5,6-dihydro-13*H*-indolo[3,2-*c*]acridine prepared by condensation of a 2,3,4,9-tetra­hydro-1*H*-carbazol-1-one with 2-amino­benzophenone. The crystals undergo a destructive phase change upon cooling at varying temperatures between 270 and 200 K, depending on cooling rate and disturbance by vibration, thus indicating supercooling of the metastable room-temperature structure at lower temperature. The overall planarity of the indolo[3,2-*c*]acridine part of the mol­ecule is inter­rupted by the saturated ethyl­ene group, and the planes of the two halves exhibit a dihedral angle of 22.05 (6)° with each other while themselves being essentially planar. Packing is dominated by C—H⋯π inter­actions. No classical hydrogen bonds or stacking inter­actions are observed.

## Related literature

For general background on the synthesis and properties of carbazole derivatives, see: Knölker & Reddy (2002 ▶); Choi *et al.* (2008 ▶). For synthesis and structures of indoloacridines, see: Sridharan *et al.* (2009*a* ▶,*b* ▶). For pharmacologically active constituents (especially carbazole alkaloids) of *Murraya koenigii* spreng, see: Iyer & Devi (2008 ▶).
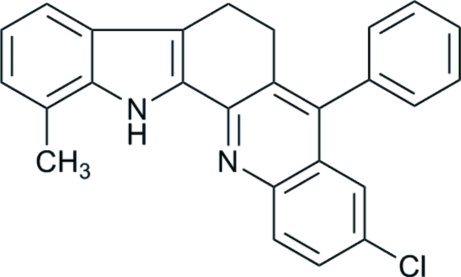


## Experimental

### 

#### Crystal data

C_26_H_19_ClN_2_
*M* *_r_* = 394.88
Triclinic, 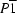

*a* = 9.981 (4) Å
*b* = 10.057 (4) Å
*c* = 10.281 (4) Å
α = 76.459 (7)°
β = 80.279 (7)°
γ = 81.754 (7)°
*V* = 983.1 (7) Å^3^
*Z* = 2
Mo *K*α radiation
μ = 0.21 mm^−1^
*T* = 293 K
0.55 × 0.20 × 0.12 mm

#### Data collection

Bruker SMART APEX CCD diffractometer
Absorption correction: multi-scan (*SADABS*; Bruker, 2008 ▶) *T*_min_ = 0.851, *T*_max_ = 0.975
10234 measured reflections
4857 independent reflections
2826 reflections with *I* > 2σ(*I*)
*R*_int_ = 0.038

#### Refinement

*R*[*F* ^2^ > 2σ(*F* ^2^)] = 0.053
*wR*(*F* ^2^) = 0.124
*S* = 1.02
4857 reflections
263 parameters
H-atom parameters constrained
Δρ_max_ = 0.17 e Å^−3^
Δρ_min_ = −0.24 e Å^−3^

### 

Data collection and cell refinement: *APEX2* (Bruker, 2008 ▶); data reduction: *SAINT*; program(s) used to solve structure: *SHELXTL* (Sheldrick, 2008 ▶); program(s) used to refine structure: *SHELXTL*; molecular graphics: *SHELXTL*; software used to prepare material for publication: *SHELXTL* and *Mercury* (Macrae *et al.*, 2008 ▶).

## Supplementary Material

Crystal structure: contains datablocks global, I. DOI: 10.1107/S1600536809013737/fl2247sup1.cif

Structure factors: contains datablocks I. DOI: 10.1107/S1600536809013737/fl2247Isup2.hkl

Additional supplementary materials:  crystallographic information; 3D view; checkCIF report

## Figures and Tables

**Table 1:** Hydrogen-bond geometry (Å, °) *Cg*1 is the centroid of the ring C1–C6 and *Cg*2 is the centroid of the indole ring.

*D*—H⋯*A*	*D*—H	H⋯*A*	*D*⋯*A*	*D*—H⋯*A*
C10—H10*B*⋯*Cg*1^i^	0.97	2.96	3.848 (3)	152
C26—H26⋯*Cg*2^i^	0.93	2.51	3.391 (3)	158

Symmetry code: (i) 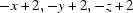.

**Fig. 1.**
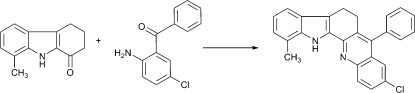
Synthesis of the title compound

**Fig. 2.**
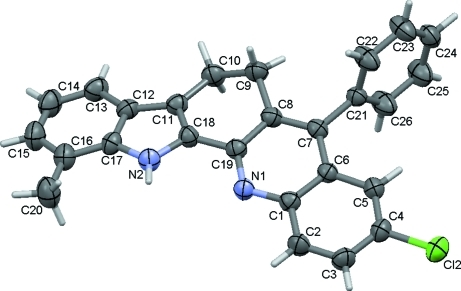
Thermal ellipsoid plot of the title compound with the atom-labeling scheme. Displacement ellipsoids are shown at the 50% probability level, H atoms are shown as capped sticks.

**Fig. 3.**
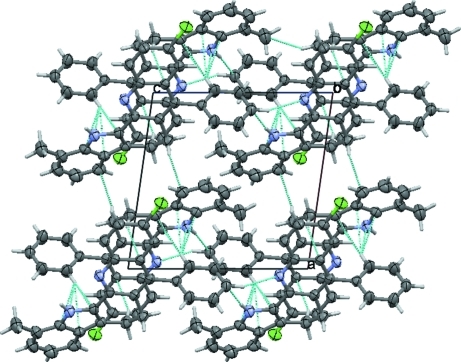
Packing view of the title compound showing C—H···π interactions and very weak C—H···C and N—H···C interactions (blue dotted lines).

**Fig. 4.**
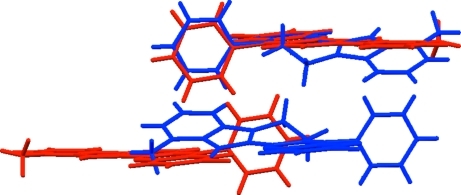
Overlay of the title compound with its hydrogenated counterpart (Sridharan et al., 2009b). The chlorobenzene part of the top molecule was used to define the overlay of the two compounds. The other molecules are created by the symmetry operations of their respective structures.

## supplementary crystallographic information

### Comment

Nitrogen-containing heterocyclic compounds are the key building blocks used to develop compounds of biological and medicinal interest to chemists. Among nitrogen heterocycles, carbazole alkaloids represent an important class of natural products. The Indian medicinal plant *Murraya koenigii* spreng (Rutaceae) has been found to be a rich source of many carbazole alkaloids (Iyer & Devi, 2008). A number of carbazole alkaloids with intriguing novel structures and useful biological activities were isolated from natural sources over the past decades which had attracted chemists to frame novel synthetic strategies towards the synthesis of carbazole and its derivatives. The continuous increase of isolable natural products as well as pharmacological action of these carbazole derivatives has generated synthetic interest; consequently the syntheses of carbazoles have been a vigorously active area of study (Knölker & Reddy, 2002, and references therein; Choi *et al.* 2008).

Based on the structural, biological and pharmacological importance of the carbazole derivatives, the present investigation was aimed to devise a viable synthetic route to prepare these classes of compound using different synthetic methodologies. For our synthetic strategy 2,3,4,9-tetra-hydro-1*H*-carbazol-1-ones, prepared in our laboratory as potential precursors, have opened new avenues for the synthesis of highly functionalized carbazole derivatives. Based on these facts we have developed and reported an efficient syntheses of novel indoloacridines and have reported the crystallographic behavior of some of these compounds (Sridharan *et al.*, 2009*a*,*b*). The current contribution presents the synthesis (Fig. 1) and crystal structure of the title compound which represents one such indoloacridine.

The compound undergoes a destructive phase change upon cooling at varying temperatures between 270 and 200 K, depending on cooling rate and disturbance by vibration, thus indicating supercooling of the room-temperature phase. To guarantee collection of a whole dataset the collection was thus performed at room temperture. An *ORTEP*style plot of the molecule is shown in Fig. 2.

The overall planarity of the indolo[3,2-*c*]acridine part of the molecule is interrupted by the saturated ethylene group of C9 and C10. The planes formed by C1 to C9, C19 and N1 as well as the plane made up of atoms C10 to C18, C20 and N2 are overall planar with r.m.s. deviations from planarity of only 0.01 and 0.03 Å, respectively. With each other the two planes form an angle of 22.05 (6)°. C10 deviates from the first plane by 0.807 (3) Å, C9 from the second by 0.476 (3) Å. The phenyl ring is at an angle to the first plane of 77.81 (6)°.

The N—H group does not form a classical hydrogen bond in the solid state and no strong π–π stacking interactions are observed. Other than van der Waals dispersive forces the packing of the compound in the solid state is dominated by C—H···π interactions (Fig. 3). The two most prominent such interactions are between C10—H10B and the centroid *Cg*1 of the ring built by atoms C1 to C6 (the chlorine-substituted phenyl ring), and between C26—H26 and the centroid *Cg*2 of the indole ring with H···*Cg* distances of 2.96 and 2.51 Å (Table 1). Additional very weak C—H···C and N—H···C interactions are indicated in Fig. 3.

In a recent publication (Sridharan *et al.*, 2009*b*) we reported the structure of the dehydrogenated derivative of the title compound. It crystallizes in a primitive inversion symmetric setting with a similar volume as for the structure of the title compound. There are however no further reaching similarities between the structures of the two compounds. The hydrogenated molecule is essentially planar and packing, shape of the unit cell and location of the inversion centers are different for the two related compounds (Fig. 4).

### Experimental

8-Methyl-2,3,4,9-tetrahydro-1*H*-carbazol-1-one (0.995 g, 5 mmol) and 2-amino-5-chlorobenzophenone (1.155 g, 5 mmol) were refluxed for 5 h in glacial acetic acid (4 ml) containing one drop of sulfuric acid. The reaction was monitored by TLC. After the completion of the reaction, the mixture was poured into crushed ice, extracted with chloroform, and the organic layer dried (Na_2_SO_4_). The crude product obtained on removal of the solvent was purified by column chromatography over silica gel using petroleum ether:ethyl acetate (98:5) to yield the title compound. 1.26 g, 64%, m.p. 527–529 K. Single crystals suitable for data collection were grown by slow evaporation from a solution in ethanol.

### Refinement

All H atoms were added in calculated positions with C—H bond distances of 0.97 (methylene), 0.93 (aromatic) and 0.96 Å (methyl) and an N—H distance of 0.86 Å. They were refined with isotropic displacement parameters *U*_iso_ of 1.5 (methyl) or 1.2 times *U*_eq_ (all others) of the adjacent C or N atom.

### Crystal data

**Table:**

C_26_H_19_ClN_2_	*Z* = 2
*M**_r_* = 394.88	*F*(000) = 412
Triclinic, *P*1	*D*_x_ = 1.334 Mg m^−^^3^
Hall symbol: -P 1	Mo *K*α radiation, λ = 0.71073 Å
*a* = 9.981 (4) Å	Cell parameters from 417 reflections
*b* = 10.057 (4) Å	θ = 2.6–30.3°
*c* = 10.281 (4) Å	µ = 0.21 mm^−^^1^
α = 76.459 (7)°	*T* = 293 K
β = 80.279 (7)°	Needle, yellow
γ = 81.754 (7)°	0.55 × 0.20 × 0.12 mm
*V* = 983.1 (7) Å^3^	

### Data collection

**Table:**

Bruker SMART APEX CCD diffractometer	4857 independent reflections
Radiation source: fine-focus sealed tube	2826 reflections with *I* > 2σ(*I*)
graphite	*R*_int_ = 0.038
ω scans	θ_max_ = 28.3°, θ_min_ = 2.1°
Absorption correction: multi-scan (*APEX2*; Bruker, 2008)	*h* = −13→13
*T*_min_ = 0.851, *T*_max_ = 0.975	*k* = −13→13
10234 measured reflections	*l* = −13→13

### Refinement

**Table:**

Refinement on *F*^2^	Primary atom site location: structure-invariant direct methods
Least-squares matrix: full	Secondary atom site location: difference Fourier map
*R*[*F*^2^ > 2σ(*F*^2^)] = 0.053	Hydrogen site location: inferred from neighbouring sites
*wR*(*F*^2^) = 0.124	H-atom parameters constrained
*S* = 1.02	*w* = 1/[σ^2^(*F*_o_^2^) + (0.0406*P*)^2^ + 0.0904*P*] where *P* = (*F*_o_^2^ + 2*F*_c_^2^)/3
4857 reflections	(Δ/σ)_max_ = 0.001
263 parameters	Δρ_max_ = 0.17 e Å^−^^3^
0 restraints	Δρ_min_ = −0.24 e Å^−^^3^

### Special details

**Table:**

Geometry. All e.s.d.'s (except the e.s.d. in the dihedral angle between two l.s. planes) are estimated using the full covariance matrix. The cell e.s.d.'s are taken into account individually in the estimation of e.s.d.'s in distances, angles and torsion angles; correlations between e.s.d.'s in cell parameters are only used when they are defined by crystal symmetry. An approximate (isotropic) treatment of cell e.s.d.'s is used for estimating e.s.d.'s involving l.s. planes.
Refinement. Refinement of *F*^2^ against ALL reflections. The weighted *R*-factor *wR* and goodness of fit *S* are based on *F*^2^, conventional *R*-factors *R* are based on *F*, with *F* set to zero for negative *F*^2^. The threshold expression of *F*^2^ > σ(*F*^2^) is used only for calculating *R*-factors(gt) *etc*. and is not relevant to the choice of reflections for refinement. *R*-factors based on *F*^2^ are statistically about twice as large as those based on *F*, and *R*- factors based on ALL data will be even larger.

### Fractional atomic coordinates and isotropic or equivalent isotropic displacement parameters (Å^2^)

**Table:**

	*x*	*y*	*z*	*U*_iso_*/*U*_eq_	
C1	1.0539 (2)	0.6878 (2)	0.9185 (2)	0.0377 (5)	
C2	1.1590 (2)	0.6123 (2)	0.8474 (2)	0.0483 (6)	
H2	1.1628	0.6231	0.7546	0.058*	
C3	1.2550 (2)	0.5237 (2)	0.9125 (2)	0.0499 (6)	
H3	1.3237	0.4741	0.8645	0.060*	
C4	1.2495 (2)	0.5079 (2)	1.0516 (2)	0.0433 (5)	
C5	1.1507 (2)	0.5793 (2)	1.1249 (2)	0.0401 (5)	
H5	1.1498	0.5674	1.2175	0.048*	
C6	1.04930 (19)	0.6717 (2)	1.05958 (19)	0.0360 (5)	
C7	0.9424 (2)	0.7498 (2)	1.12968 (19)	0.0366 (5)	
C8	0.8504 (2)	0.8392 (2)	1.0570 (2)	0.0384 (5)	
C9	0.7306 (2)	0.9240 (2)	1.1197 (2)	0.0481 (6)	
H9A	0.7504	0.9349	1.2056	0.058*	
H9B	0.6509	0.8741	1.1379	0.058*	
C10	0.6961 (2)	1.0667 (2)	1.0325 (2)	0.0481 (6)	
H10A	0.6064	1.1058	1.0675	0.058*	
H10B	0.7625	1.1270	1.0357	0.058*	
C11	0.6973 (2)	1.0559 (2)	0.8900 (2)	0.0399 (5)	
C12	0.6403 (2)	1.1467 (2)	0.7787 (2)	0.0406 (5)	
C13	0.5656 (2)	1.2770 (2)	0.7608 (2)	0.0509 (6)	
H13	0.5405	1.3216	0.8323	0.061*	
C14	0.5304 (2)	1.3371 (3)	0.6356 (3)	0.0607 (7)	
H14	0.4825	1.4245	0.6219	0.073*	
C15	0.5649 (2)	1.2703 (3)	0.5283 (3)	0.0606 (7)	
H15	0.5364	1.3135	0.4457	0.073*	
C16	0.6396 (2)	1.1428 (2)	0.5396 (2)	0.0496 (6)	
C17	0.6786 (2)	1.0840 (2)	0.6669 (2)	0.0412 (5)	
C18	0.7711 (2)	0.9486 (2)	0.8411 (2)	0.0388 (5)	
C19	0.8654 (2)	0.8472 (2)	0.91467 (19)	0.0370 (5)	
C20	0.6795 (3)	1.0715 (3)	0.4238 (2)	0.0692 (8)	
H20A	0.6358	1.1226	0.3482	0.104*	
H20B	0.6512	0.9805	0.4503	0.104*	
H20C	0.7769	1.0657	0.3987	0.104*	
C21	0.9294 (2)	0.7292 (2)	1.27990 (19)	0.0373 (5)	
C22	0.8344 (2)	0.6490 (3)	1.3624 (2)	0.0557 (6)	
H22	0.7773	0.6089	1.3236	0.067*	
C23	0.8222 (3)	0.6269 (3)	1.5009 (2)	0.0594 (7)	
H23	0.7574	0.5723	1.5548	0.071*	
C24	0.9056 (2)	0.6854 (2)	1.5591 (2)	0.0493 (6)	
H24	0.8983	0.6703	1.6527	0.059*	
C25	0.9988 (3)	0.7656 (3)	1.4794 (2)	0.0608 (7)	
H25	1.0548	0.8065	1.5188	0.073*	
C26	1.0116 (2)	0.7874 (3)	1.3397 (2)	0.0575 (7)	
H26	1.0767	0.8420	1.2864	0.069*	
Cl2	1.37259 (6)	0.39220 (7)	1.13247 (6)	0.0601 (2)	
N1	0.96084 (17)	0.77544 (17)	0.84684 (16)	0.0407 (4)	
N2	0.75957 (17)	0.96338 (17)	0.70665 (17)	0.0433 (4)	
H2A	0.7966	0.9069	0.6563	0.052*	

### Atomic displacement parameters (Å^2^)

**Table:**

	*U*^11^	*U*^22^	*U*^33^	*U*^12^	*U*^13^	*U*^23^
C1	0.0383 (11)	0.0386 (12)	0.0376 (11)	−0.0026 (9)	−0.0047 (9)	−0.0122 (9)
C2	0.0522 (13)	0.0554 (15)	0.0375 (12)	0.0053 (11)	−0.0065 (10)	−0.0170 (11)
C3	0.0457 (13)	0.0552 (15)	0.0493 (13)	0.0090 (11)	−0.0070 (10)	−0.0208 (12)
C4	0.0414 (12)	0.0400 (12)	0.0476 (13)	0.0002 (10)	−0.0110 (10)	−0.0069 (10)
C5	0.0420 (12)	0.0422 (13)	0.0352 (11)	−0.0035 (10)	−0.0069 (9)	−0.0059 (10)
C6	0.0382 (11)	0.0361 (11)	0.0348 (11)	−0.0046 (9)	−0.0044 (9)	−0.0098 (9)
C7	0.0406 (11)	0.0359 (12)	0.0335 (10)	−0.0060 (9)	−0.0032 (9)	−0.0084 (9)
C8	0.0400 (11)	0.0383 (12)	0.0363 (11)	−0.0038 (9)	−0.0015 (9)	−0.0098 (9)
C9	0.0495 (13)	0.0506 (14)	0.0398 (12)	0.0051 (11)	−0.0006 (10)	−0.0109 (11)
C10	0.0538 (14)	0.0446 (14)	0.0448 (13)	0.0035 (11)	−0.0038 (10)	−0.0147 (11)
C11	0.0397 (11)	0.0359 (12)	0.0427 (12)	−0.0005 (9)	−0.0038 (9)	−0.0094 (10)
C12	0.0360 (11)	0.0391 (12)	0.0455 (12)	−0.0003 (10)	−0.0040 (9)	−0.0105 (10)
C13	0.0504 (14)	0.0418 (13)	0.0583 (15)	0.0047 (11)	−0.0051 (11)	−0.0139 (12)
C14	0.0621 (16)	0.0425 (14)	0.0703 (17)	0.0089 (12)	−0.0152 (13)	−0.0026 (13)
C15	0.0665 (16)	0.0538 (16)	0.0576 (15)	0.0043 (13)	−0.0236 (13)	−0.0002 (13)
C16	0.0544 (14)	0.0459 (14)	0.0491 (14)	−0.0024 (11)	−0.0170 (11)	−0.0064 (11)
C17	0.0403 (12)	0.0358 (12)	0.0474 (13)	−0.0007 (10)	−0.0109 (9)	−0.0076 (10)
C18	0.0438 (12)	0.0377 (12)	0.0352 (11)	−0.0016 (10)	−0.0073 (9)	−0.0090 (9)
C19	0.0402 (11)	0.0356 (12)	0.0361 (11)	−0.0023 (9)	−0.0048 (9)	−0.0106 (9)
C20	0.090 (2)	0.0684 (18)	0.0523 (15)	0.0027 (15)	−0.0249 (14)	−0.0146 (14)
C21	0.0389 (11)	0.0392 (12)	0.0330 (11)	−0.0012 (10)	−0.0040 (9)	−0.0089 (9)
C22	0.0668 (16)	0.0670 (17)	0.0387 (13)	−0.0280 (14)	−0.0050 (11)	−0.0115 (12)
C23	0.0729 (17)	0.0674 (17)	0.0386 (13)	−0.0267 (14)	0.0006 (12)	−0.0069 (12)
C24	0.0595 (15)	0.0525 (14)	0.0338 (11)	0.0004 (12)	−0.0067 (10)	−0.0087 (11)
C25	0.0627 (16)	0.0808 (19)	0.0467 (14)	−0.0232 (15)	−0.0142 (12)	−0.0151 (13)
C26	0.0587 (15)	0.0747 (18)	0.0430 (13)	−0.0280 (14)	−0.0049 (11)	−0.0096 (13)
Cl2	0.0529 (4)	0.0598 (4)	0.0614 (4)	0.0131 (3)	−0.0138 (3)	−0.0083 (3)
N1	0.0444 (10)	0.0417 (10)	0.0368 (9)	0.0045 (8)	−0.0088 (8)	−0.0134 (8)
N2	0.0524 (11)	0.0383 (10)	0.0408 (10)	0.0047 (9)	−0.0107 (8)	−0.0148 (8)

### Geometric parameters (Å, °)

**Table:**

C1—N1	1.369 (2)	C13—H13	0.9300
C1—C2	1.408 (3)	C14—C15	1.393 (3)
C1—C6	1.415 (3)	C14—H14	0.9300
C2—C3	1.361 (3)	C15—C16	1.378 (3)
C2—H2	0.9300	C15—H15	0.9300
C3—C4	1.394 (3)	C16—C17	1.400 (3)
C3—H3	0.9300	C16—C20	1.499 (3)
C4—C5	1.362 (3)	C17—N2	1.375 (2)
C4—Cl2	1.742 (2)	C18—N2	1.378 (2)
C5—C6	1.416 (3)	C18—C19	1.450 (3)
C5—H5	0.9300	C19—N1	1.315 (2)
C6—C7	1.428 (3)	C20—H20A	0.9600
C7—C8	1.375 (3)	C20—H20B	0.9600
C7—C21	1.495 (3)	C20—H20C	0.9600
C8—C19	1.430 (3)	C21—C26	1.368 (3)
C8—C9	1.509 (3)	C21—C22	1.377 (3)
C9—C10	1.530 (3)	C22—C23	1.375 (3)
C9—H9A	0.9700	C22—H22	0.9300
C9—H9B	0.9700	C23—C24	1.368 (3)
C10—C11	1.492 (3)	C23—H23	0.9300
C10—H10A	0.9700	C24—C25	1.355 (3)
C10—H10B	0.9700	C24—H24	0.9300
C11—C18	1.364 (3)	C25—C26	1.388 (3)
C11—C12	1.432 (3)	C25—H25	0.9300
C12—C13	1.400 (3)	C26—H26	0.9300
C12—C17	1.410 (3)	N2—H2A	0.8600
C13—C14	1.369 (3)		
			
N1—C1—C2	118.08 (18)	C13—C14—H14	119.3
N1—C1—C6	122.72 (18)	C15—C14—H14	119.3
C2—C1—C6	119.20 (18)	C16—C15—C14	122.7 (2)
C3—C2—C1	121.0 (2)	C16—C15—H15	118.6
C3—C2—H2	119.5	C14—C15—H15	118.6
C1—C2—H2	119.5	C15—C16—C17	115.4 (2)
C2—C3—C4	119.4 (2)	C15—C16—C20	123.0 (2)
C2—C3—H3	120.3	C17—C16—C20	121.6 (2)
C4—C3—H3	120.3	N2—C17—C16	128.9 (2)
C5—C4—C3	121.83 (19)	N2—C17—C12	108.05 (17)
C5—C4—Cl2	119.76 (16)	C16—C17—C12	123.0 (2)
C3—C4—Cl2	118.41 (16)	C11—C18—N2	110.36 (18)
C4—C5—C6	119.80 (18)	C11—C18—C19	123.91 (18)
C4—C5—H5	120.1	N2—C18—C19	124.99 (18)
C6—C5—H5	120.1	N1—C19—C8	125.10 (18)
C1—C6—C5	118.68 (18)	N1—C19—C18	118.81 (18)
C1—C6—C7	118.34 (18)	C8—C19—C18	116.00 (18)
C5—C6—C7	122.98 (18)	C16—C20—H20A	109.5
C8—C7—C6	118.72 (17)	C16—C20—H20B	109.5
C8—C7—C21	121.44 (18)	H20A—C20—H20B	109.5
C6—C7—C21	119.80 (17)	C16—C20—H20C	109.5
C7—C8—C19	118.02 (18)	H20A—C20—H20C	109.5
C7—C8—C9	123.77 (18)	H20B—C20—H20C	109.5
C19—C8—C9	118.14 (18)	C26—C21—C22	117.98 (19)
C8—C9—C10	114.34 (18)	C26—C21—C7	121.54 (19)
C8—C9—H9A	108.7	C22—C21—C7	120.48 (18)
C10—C9—H9A	108.7	C23—C22—C21	121.5 (2)
C8—C9—H9B	108.7	C23—C22—H22	119.3
C10—C9—H9B	108.7	C21—C22—H22	119.3
H9A—C9—H9B	107.6	C24—C23—C22	119.8 (2)
C11—C10—C9	109.85 (18)	C24—C23—H23	120.1
C11—C10—H10A	109.7	C22—C23—H23	120.1
C9—C10—H10A	109.7	C25—C24—C23	119.5 (2)
C11—C10—H10B	109.7	C25—C24—H24	120.3
C9—C10—H10B	109.7	C23—C24—H24	120.3
H10A—C10—H10B	108.2	C24—C25—C26	120.7 (2)
C18—C11—C12	106.56 (18)	C24—C25—H25	119.6
C18—C11—C10	121.04 (18)	C26—C25—H25	119.6
C12—C11—C10	132.23 (19)	C21—C26—C25	120.5 (2)
C13—C12—C17	118.90 (19)	C21—C26—H26	119.7
C13—C12—C11	134.2 (2)	C25—C26—H26	119.7
C17—C12—C11	106.81 (18)	C19—N1—C1	117.09 (17)
C14—C13—C12	118.4 (2)	C17—N2—C18	108.15 (17)
C14—C13—H13	120.8	C17—N2—H2A	125.9
C12—C13—H13	120.8	C18—N2—H2A	125.9
C13—C14—C15	121.4 (2)		
			
N1—C1—C2—C3	179.8 (2)	C15—C16—C17—C12	2.6 (3)
C6—C1—C2—C3	0.5 (3)	C20—C16—C17—C12	−178.5 (2)
C1—C2—C3—C4	−0.2 (3)	C13—C12—C17—N2	175.20 (18)
C2—C3—C4—C5	−0.3 (3)	C11—C12—C17—N2	−2.2 (2)
C2—C3—C4—Cl2	179.24 (18)	C13—C12—C17—C16	−3.4 (3)
C3—C4—C5—C6	0.6 (3)	C11—C12—C17—C16	179.2 (2)
Cl2—C4—C5—C6	−178.99 (15)	C12—C11—C18—N2	−2.4 (2)
N1—C1—C6—C5	−179.55 (18)	C10—C11—C18—N2	−178.18 (18)
C2—C1—C6—C5	−0.2 (3)	C12—C11—C18—C19	168.12 (19)
N1—C1—C6—C7	0.3 (3)	C10—C11—C18—C19	−7.7 (3)
C2—C1—C6—C7	179.7 (2)	C7—C8—C19—N1	0.0 (3)
C4—C5—C6—C1	−0.3 (3)	C9—C8—C19—N1	−177.1 (2)
C4—C5—C6—C7	179.8 (2)	C7—C8—C19—C18	−176.48 (18)
C1—C6—C7—C8	−1.3 (3)	C9—C8—C19—C18	6.4 (3)
C5—C6—C7—C8	178.57 (19)	C11—C18—C19—N1	−159.1 (2)
C1—C6—C7—C21	176.41 (18)	N2—C18—C19—N1	10.0 (3)
C5—C6—C7—C21	−3.7 (3)	C11—C18—C19—C8	17.6 (3)
C6—C7—C8—C19	1.1 (3)	N2—C18—C19—C8	−173.25 (19)
C21—C7—C8—C19	−176.52 (18)	C8—C7—C21—C26	−103.4 (3)
C6—C7—C8—C9	178.06 (19)	C6—C7—C21—C26	78.9 (3)
C21—C7—C8—C9	0.4 (3)	C8—C7—C21—C22	77.4 (3)
C7—C8—C9—C10	144.9 (2)	C6—C7—C21—C22	−100.2 (2)
C19—C8—C9—C10	−38.2 (3)	C26—C21—C22—C23	−0.3 (4)
C8—C9—C10—C11	45.2 (3)	C7—C21—C22—C23	178.9 (2)
C9—C10—C11—C18	−24.0 (3)	C21—C22—C23—C24	0.0 (4)
C9—C10—C11—C12	161.5 (2)	C22—C23—C24—C25	0.5 (4)
C18—C11—C12—C13	−174.0 (2)	C23—C24—C25—C26	−0.8 (4)
C10—C11—C12—C13	1.1 (4)	C22—C21—C26—C25	0.0 (4)
C18—C11—C12—C17	2.8 (2)	C7—C21—C26—C25	−179.2 (2)
C10—C11—C12—C17	177.9 (2)	C24—C25—C26—C21	0.6 (4)
C17—C12—C13—C14	1.3 (3)	C8—C19—N1—C1	−1.0 (3)
C11—C12—C13—C14	177.8 (2)	C18—C19—N1—C1	175.42 (17)
C12—C13—C14—C15	1.3 (4)	C2—C1—N1—C19	−178.57 (19)
C13—C14—C15—C16	−2.1 (4)	C6—C1—N1—C19	0.8 (3)
C14—C15—C16—C17	0.1 (4)	C16—C17—N2—C18	179.2 (2)
C14—C15—C16—C20	−178.7 (2)	C12—C17—N2—C18	0.8 (2)
C15—C16—C17—N2	−175.6 (2)	C11—C18—N2—C17	1.1 (2)
C20—C16—C17—N2	3.2 (4)	C19—C18—N2—C17	−169.34 (19)

### Hydrogen-bond geometry (Å, °)

**Table:**

*D*—H···*A*	*D*—H	H···*A*	*D*···*A*	*D*—H···*A*
C10—H10B···Cg1^i^	0.97	2.96	3.848 (3)	152
C26—H26···Cg2^i^	0.93	2.51	3.391 (3)	158

Symmetry codes: (i) −*x*+2, −*y*+2, −*z*+2.
